# Draft Genome of *Thermanaerothrix daxensis* GNS-1, a Thermophilic Facultative Anaerobe from the *Chloroflexi* Class *Anaerolineae*

**DOI:** 10.1128/genomeA.01354-15

**Published:** 2015-11-19

**Authors:** Laura A. Pace, James Hemp, Lewis M. Ward, Woodward W. Fischer

**Affiliations:** a Department of Medicine, University of California San Diego, La Jolla, California, USA; b Division of Geological and Planetary Sciences, California Institute of Technology, Pasadena, California, USA

## Abstract

We present the draft genome of *Thermanaerothrix daxensis* GNS-1, a thermophilic member of the *Chloroflexi* phylum. This organism was initially characterized as a nonmotile, strictly anaerobic fermenter; however, genome analysis demonstrates that it encodes genes for a flagellum and multiple pathways for aerobic and anaerobic respiration.

## GENOME ANNOUNCEMENT

*Thermanaerothrix daxensis* GNS-1 was isolated from a deep groundwater aquifer (149 m) housed within sedimentary strata of the large Mesozoic and Tertiary Aquitaine Basin in southwestern France (1). Closely related strains have been reported from a hot spring in Yellowstone National Park (2), a hot spring in southwestern Taiwan, geothermal soil, a thermophilic anaerobic digestive sludge, and a thermophilic electrochemical cell (3). *T. daxensis* is a filamentous, nonsporulating organism that can ferment a number of sugars and organic acids (1). It grows optimally at 65°C (range 50 to 73°C) and pH 7 (range pH 5.8 to 8.5) (1).

The genome of *Thermanaerothrix daxensis* GNS-1 (DSM 23592) was sequenced as part of a project to expand the phylogenetic breadth of *Chloroflexi* genomes. Genome sequencing was performed at Seqmatic using the Illumina MiSeq sequencing platform. SPAdes version 3.1.1 (4) was used to assemble the genome. The genome was screened for contaminants based on sequence coverage, GC composition, and BLAST hits of conserved single-copy genes. Genome annotation was performed using the NCBI Prokaryotic Genome Annotation Pipeline. The draft genome is 3.06 Mb in size, assembled into 6 contigs. It encodes 2,798 genes, 2,395 coding sequences, 1 16S RNA, 47 tRNAs, and 4 CRISPR arrays. It is estimated to be ~95% complete based on conserved single-copy genes (106/111).

Genome analysis of *T. daxensis* detected the presence of aerobic and anaerobic respiration pathways, hinting at a richer physiology than previously recognized. It encodes for Complex I (NADH dehydrogenase), Complex II (succinate dehydrogenase), and an aerobic CO dehydrogenase. It also has two aerobic respiration modules; an A-family heme-copper oxygen reductase coupled to an alternative complex III (ACIII) (5), and a quinol *bd* oxidase (6). In addition, *T. daxensis* has two respiratory nitrite reductases; NirS, which reduces NO_2_^−^ to NO, and NrfA that reduces NO_2_^−^ to NH_4_^+^. The genome provides no evidence for the presence of LPS biosynthesis genes or outer membrane proteins, suggesting that this organism has only one membrane (7). Furthermore, it encodes for a Gram-positive flagella and is likely motile under certain physiological conditions.

### Nucleotide sequence accession number.

This whole-genome shotgun project has been deposited in DDBJ/EMBL/GenBank under the accession number LGKO00000000.
